# Biochemical characteristics of extracts from proallergenic microfungi *Erysiphe Palczewskii* and *Erysiphe convolvuli*

**DOI:** 10.1186/s12864-025-11862-w

**Published:** 2025-08-18

**Authors:** Małgorzata Pac-Sosińska, Marta Palusińska-Szysz, Monika Sztandera-Tymoczek, Urszula Świderska, Sylwia Wdowiak-Wróbel, Agata Malinowska, Bianka Świderska, Agnieszka Szuster-Ciesielska

**Affiliations:** 1 Department of Virology and Immunology, Institute of Biological Sciences, Maria Curie-Skłodowska University, Akademicka 19, Lublin, 20-033 Poland; 2 Department of Genetics and Microbiology, Institute of Biological Sciences, Maria Curie-Skłodowska University, Lublin, Poland; 3 Department of Botany, Mycology and Ecology, Institute of Biological Sciences, Maria Curie-Skłodowska University, Lublin, Poland; 4 Mass Spectrometry Laboratory, Institute of Biochemistry and Biophysics, Polish Academy of Sciences, Warsaw, Poland

**Keywords:** Plant microfungi, *Erysiphe Palczewskii*, *E. convolvuli*, Proteomics, Fatty acids, Carbohydrates

## Abstract

**Background:**

Phytopathogenic microfungi *Erysiphe palczewskii* and *Erysiphe convolvuli*, are ectoparasites causing powdery mildew in common plants. Fungi often produce immunogenic factors triggering allergic reactions, with airborne protein allergens playing a pivotal role in respiratory allergies. This study delves into the biochemical characterization of *E. palczewskii* and *E. convolvuli*, focusing on their potential allergenic properties.

**Methods:**

The composition of the tested fungi’s fatty acids and sugars was analyzed using liquid chromatography coupled with mass spectrometry (LC/MS). Protein extracts were also subjected to liquid chromatography-mass spectrometry analysis (LC/MS).

**Results:**

The proteomic analysis of *E. palczewskii* identified 1118 peptides, with 68.8% unique to this species. The abundant proteins included ribosomal proteins, heat shock proteins, enolase, fumarate reductase, and nucleoside diphosphate kinase. The *E. convolvuli* analysis revealed 770 peptides, with 47% unique sequences. The abundant proteins included ribosomal proteins, heat shock proteins, NDPK, glycerol dehydrogenase, malate dehydrogenase, and a Rho GDP dissociation inhibitor. The analysis of the fatty acid composition revealed that both species exhibited a diverse profile and synthesized fatty acid 18:2, which constituted approximately 30% of the total fatty acids in *E. palczewskii*. The analyzed fungi primarily produced hexoses, pentoses, and hexosamines.

**Conclusion:**

The comparative proteomic analysis provided insights into the unique and shared proteins of *E. palczewskii* and *E. convolvuli*. Several proteins, including heat shock proteins and enzymes involved in metabolic processes, exhibited allergenic potential. The studied fungi contained a high concentration of fatty acid 18:2, a precursor of arachidonic acid, which is involved in developing inflammatory responses.

**Supplementary Information:**

The online version contains supplementary material available at 10.1186/s12864-025-11862-w.

## Background

Fungi often serve as a significant source of immunogenic factors that can elicit allergic reactions in humans. Due to their mode of dissemination, fungal allergens constitute an essential factor in developing respiratory allergies [1, 2]. More than 180 fungal species have been shown to produce allergenic proteins, with those of the Ascomycota and Basidiomycota phyla being clinically important. It has been estimated that the most thoroughly studied allergenic fungi have up to 20 well-characterized allergens and additional 27 to 60 less well-characterized IgE-binding proteins [3].

Most fungal allergens are proteins or glycoproteins, but mannans from *Candida albicans* and *Malassezia furfur* may also be allergenic [4]. Allergens may be cell wall- or cytoplasm-derived. Many are involved in protein synthesis or energy metabolism, although secretory (e.g., proteases) allergens also may be involved. Examples of common allergens across fungal species (both *Ascomycota* and *Basidiomycota*) include enolases, heat shock proteins (HSPs), aldehyde dehydrogenases, thioredoxins, proteases, and cyclophilins (peptidyl-prolyl isomerase). However, they may have minor or major significance, depending on the species [3].

The plant pathogenic microascomycetes of the genus *Erysiphe* (Leotiomycetes, Helotiales), such as *E. palczewskii* and *E. convolvuli*, cause a disease commonly referred to as powdery mildew. *E. palczewskii* infects the Siberian pea-tree (*Caragana arborescens*), while *E. convolvuli* is a pathogen of the field bindweed (*Convolvulus arvensis*). Due to their frequent occurrence in the anthropic environment, they have also been investigated for their potential threat to human health, particularly with reference to their proallergenic activity [5], which makes the identification and characterization of specific allergens produced by these microorganisms a crucial issue.

Therefore, this study aimed to conduct a comparative analysis of the protein, fatty acid (FA), and carbohydrate profiles of *E. palczewskii* and *E. convolvuli*, with a particular focus on their allergenic potential. The knowledge of the protein profiles of these microascomycetes is fundamental for understanding their pathogenic mechanisms and identifying potential allergens that may trigger allergic reactions in humans. These analyses can provide valuable information about the uniqueness of proteins characteristic of each species and help evaluate which proteins may serve as potential biomarkers. These proteins may be present in such fungal structures as spores, mycelium, or other fragments, posing a potential risk to sensitive individuals.

## Results

The proteomics of fungi in the *Leotiomycetes* class, to which the genus *Erysiphe* belongs, has been extensively studied. The Mascot database contains approximately 883,173 characterized peptide sequences. This extensive database has facilitated the effective and precise identification of proteins in the samples from *E. palczewskii* and *E. convolvuli*.

### Proteomic analysis of erysiphe Palczewskii

The proteomic analysis of *E. palczewskii* facilitated the identification of proteins produced by this fungus and those responsible for the allergenic potential that may contribute to human immune responses. The characterization of sequences derived from conidia led to the identification of 1118 peptide sequences. The comparison of the protein profiles of both strains revealed that 68.8% (637 sequences) of all the peptides from *E. palczewskii* are unique. In comparison, 31.2% (289 sequences) were present in both *E. palczewskii* and *E. convolvuli*.

Subsequently, the peptides unique to *E. palczewskii* were characterized in the entire genus *Erysiphe*. These studies identified 412 sequences found only in *E*. *palczewskii* (about 44.5% of all hypothetical proteins).

**Fig. 1:**
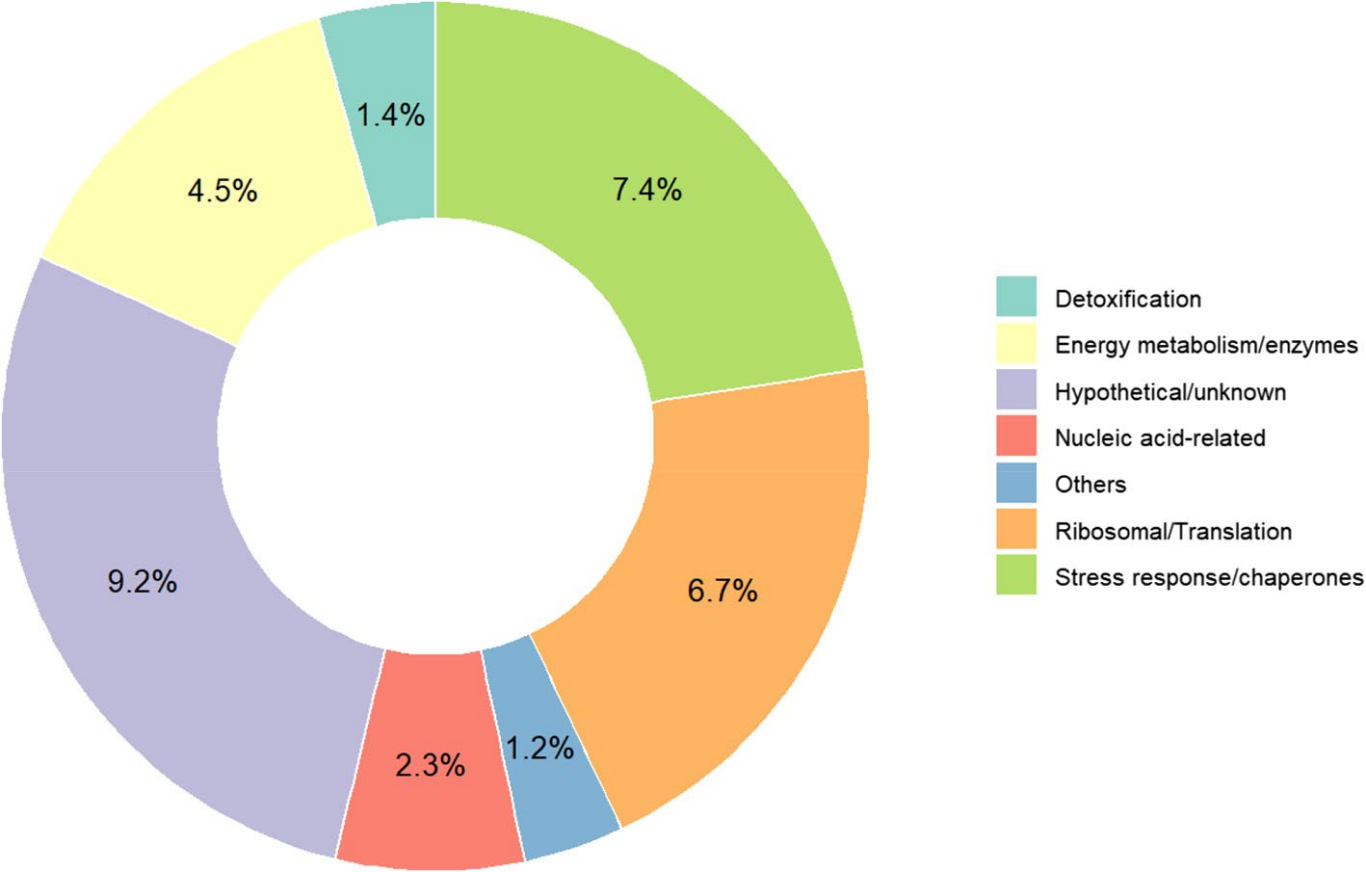
Functional classification of the most abundant proteins identified in *E. palczewskii*. The donut chart illustrates the relative abundance of proteins categorized by their predicted or known biological functions. Percentages indicate the proportion of each functional category among all identified sequences

The functional classification of the most abundant proteins reveals that a substantial portion (9.2%) corresponds to hypothetical or uncharacterized proteins, indicating limited current knowledge about their roles. Ribosomal and translation-related proteins constitute 7.4% of the identified peptides, closely followed by those involved in stress response and chaperone functions (6.7%), energy metabolism (4.5%), and detoxification (1.4%). These results are summarized in Fig. 1.

Among the most abundantly occurring proteins, various ribosomal proteins (e.g., ubiquitin-40s S31 protein), HSP (60 kDa, 70 kDa, 90 kDa), enolase, fumarate reductase, trypsinogen precursors, and nucleoside diphosphate kinase were identified (Table 1).

**Table 1:** Most abundant proteins identified in *Erysiphe palczewskii* (n.d.– no data)

**N**^**o**^	**Peptide**	**Sequence frequency**	**Percentage among all sequences**	**Common name of target**	**Length [aa]**	**Molecular weight [da]**	**Coverage [%]**	**Characterized in**	**References**
1.	POS88135.1	158	3.95	Ribosomal protein S5 2-like protein/ heat shock protein	687	77 867	15	*Erysiphe pulchra*	[76]
2.	KHJ33942.1	109	2.73	putative ubiquitin-40s ribosomal protein s31 fusion protein	155	17 883	30	*E. necator*	[77]
3.	EPQ66886.1	108	2.7	hypothetical protein BGT96224_2407	155	17 894	30	*Blumeria graminis f. sp. tritici*	[78]
4.	RKF58703.1	105	2.63	Heat shock 70 kDa protein	647	70 592	10	*E. neolycopersici*	[79]
5.	EPQ67014.1	84	2.1	Enolase	438	47 161	19	*B. graminis f. sp. tritici 96224*	[78]
6.	KHJ32358.1	77	1.93	putative heat shock protein 90	702	79 778	11	*E. necator*	[77]
7.	RKF55285.1	62	1.55	n.d	n.d	n.d	n.d	n.d	n.d
8.	KHJ31784.1	59	1.48	putative heat shock protein 90	353	38 636	6	*E. necator*	[77]
9.	POS84163.1	58	1.45	hypothetical protein EPUL_004543	155	16 258	17	*E. pulchra*	[76]
10.	RKF53964.1	57	1.43	Glutathione S-transferase hmp2	212	24 369	20	*E. neolycopersici*	[76]
11.	KHJ33155.1	53	1.33	putative heat shock protein 60	585	61 931	12	*E. necator*	[76]
12.	RKF65990.1	51	1.28	Fumarate reductase	632	68 809	13	*E. neolycopersici*	[76]
13.	RKF64955.1	49	1.23	Nucleoside diphosphate kinase	195	21 818	14	*E. neolycopersici [O. neolycopersici]*	[76]
14.	RKF58605.1	47	1.22	n.d	n.d	n.d	n.d	n.d	[76]
15.	RKF59869.1	46	1.15	putative iswi chromatin-remodeling complex atpase isw2	1 660	188 015	4	*E. neolycopersici*	[76]
16.	POS84429.1	46	1.15	enolase	438	47 427	15	*E. pulchra*	[76]
17.	RKF54333.1	46	1.15	putative 5-methyltetrahydropteroyltriglutamate-homocysteine methyltransferase	767	86 449	3	*Golovinomyces cichoracearum*	[79]
18.	RKF61509.1	45	1.13	hypothetical protein OnM2_041055	200	22 667	15	*E. neolycopersici*	[79]
19.	EPQ65210.1	45	1.13	hypothetical protein BGT96224_929	268	30 124	27	*B. graminis f. sp. tritici* 96224	[78]

The most frequently identified protein was the ribosomal protein S5 2-like (POS88135.1), which also functions as a heat shock protein. Peptides derived from this protein were detected 158 times, accounting for 3.95% of all assigned peptide spectra. The ribosomal fusion protein S31, which forms part of the 40 S subunit and is conjugated with ubiquitin (KHJ33942.1), constitutes 2.73% of the entire proteome. This protein comprises 155 amino acids with a molecular mass of 17.883 kDa. Its involvement in ribosomal activity and ubiquitination suggests a role in protein synthesis and regulation of cellular stress responses. A similar peptide has previously been characterized in *Erysiphe necator*, highlighting its potential significance in fungal biology and functional conservation across species [6–9].

A hypothetical 155-amino-acid peptide, BGT96224_2407 (EPQ66886.1), accounts for 2.7% of the proteome. It has a molecular mass of 17.894 kDa, and its exact function remains uncharacterized, but its prevalence within the proteome suggests it could play a significant role in cellular processes. This peptide shows structural similarity to a counterpart found in *Blumeria graminis* f. sp. *tritici* (formerly *Erysiphe graminis*), indicating potential functional similarities between these fungal species, particularly concerning pathogen-host interactions [10–12].

### Heat shock proteins

In the *E. palczewskii* preparations, a large number of proteins associated with the heat shock response were identified. HSP play a crucial role in fungi, performing diverse functions essential for cellular homeostasis and stress response. These proteins respond to environmental changes, regulate morphogenesis, contribute to antifungal drug resistance, and influence pathogenicity. The most abundantly detected HSPs in *E. palczewskii* were as follows:

- HSP70 (RKF58703.1) constitutes 2.63% of the total proteome and comprises 647 amino acids. HSP70 is well-documented in allergen databases. Due to its role in protein folding, cellular repair, and stress response, HSP70 is frequently implicated in immune responses and allergic reactions. It is a key protein in fungal allergenicity studies [6, 7].
- HSP60 (EKP91788.1) represents 2.6% of the proteome and comprises 551 amino acids. This highly conserved protein is classified as a mold allergen, mainly associated with allergic asthma. Its expression is upregulated after exposure to mold fungi, suggesting that it may function not only as an allergen but also as a co-factor that exacerbates allergic symptoms [6, 7].
- HSP90 (BHH54667.1) comprises 2.4% of the proteome. It is a 724-amino acid chaperone protein involved in adequate folding and stabilizing other proteins, and it regulates critical signaling pathways that promote cell survival in stress conditions. Beyond its role in cellular stress responses, HSP90 is also implicated in regulating immune responses and can contribute to the induction of allergic reactions, emphasizing its significance in allergy-related pathologies [6, 7].

The proteomic analysis also identified several proteins and peptides present in *E. palczewskii* but absent in *E. convolvuli*, as detailed in Table 2. The most abundant of these proteins are as follows:

**Table 2:** Most unique proteins identified in *Erysiphe palczewskii*

**N**^**o**^	**Peptide**	**Common name of target**	**Length (aa)**	**Molecular weight**	**Coverage [%]**	**Characterized in**	**References**
1.	EPQ66886.1	hypothetical protein BGT96224_2407	155	17 894	10	*B. graminis f.* sp*. tritici *96224	[78]
2.	POS84163.1	hypothetical protein EPUL_004543	155	16 258	17	*E. pulchra*	[76]
3.	POS84429.1	enolase	438	47 427	15	*E. pulchra*	[76]
4.	RKF54333.1	putative 5-methyltetrahydropteroyltriglutamate-homocysteine methyltransferase	767	86 449	3	*G. cichoracearum*	[79]
5.	RKF53920.1	Transaldolase	335	36 875	8	*E.neolycopersici (O. neolycopersici)*	[79]
6.	KHJ32860.1	putative 14-3-3 protein	245	27 614	10	*E. necator*	[77]
7.	RKF64822.1	putative 5-methyltetrahydropteroyltriglutamate-homocysteine methyltransferase	767	86 741	3	*E. neolycopersici*	[79]
8.	POS88442.1	catalase-domain-containing protein, partial	741	84 120	4	*E. pulchra*	[76]
9.	POS83041.1	triosephosphate isomerase, partial	238	26 118	5	*E. pulchra*	[76]
10.	POS87081.1	putative acyl-CoA dehydrogenase	546	61 249	5	*E. pulchra*	[76]

- Transaldolase (RKF53920.1) is an enzyme consisting of 335 amino acids with a molecular weight of 35.875 kDa. It is involved in the pentose phosphate pathway and plays a role in cellular metabolism [6].
- Triosephosphate isomerase (TPI or TIM) (POS83041.1) is a key enzyme in glycolysis catalyzing the interconversion of dihydroxyacetone phosphate (DHAP) and glyceraldehyde-3-phosphate (G3P), both of which are vital intermediates in cellular energy production. A partial sequence of this enzyme, with 238 amino acids and a molecular mass of 26.118 kDa, was identified in *E. palczewskii* [13].
- Acyl-CoA dehydrogenase (POS87081.1) is composed of 546 amino acids with a molecular weight of 61.249 kDa. This enzyme is involved in FA metabolism and plays an essential role in energy production and cellular homeostasis [13].
- Hypothetical protein 14-3-3 (KHJ32860.1) consists of 245 amino acids and has a molecular mass of 27.614 kDa. It has an unknown function but may be involved in signaling pathways or stress response mechanisms. Its role in fungal pathogenesis and allergenicity requires further investigation [6, 13].

The characterization of these proteins proved to be challenging due to the limited representation of *E. palczewskii* and related organisms in the available databases. The lack of detailed references in the existing literature and databases further hindered a more precise annotation of these proteins. As a result, the proteins were identified primarily through analogy with other previously characterized proteins from the genus *Erysiphe* and the order *Erysiphales*. These findings emphasize the need for further investigation to better understand the specific roles of these proteins in the pathogenesis of allergies caused by *E. palczewskii.*

#### Proteomic analysis of erysiphe convolvuli

The proteomic analysis of *E. convolvuli* identified 770 protein sequences. Of these, 362 (47%) were unique to *E. convolvuli* and not found in *E. palczewskii*, while 359 peptides were common to both fungi. Some peptides were characterized for the first time and do not have references in databases for other species (Table 3).

**Table 3:** Most abundant proteins identified in *Erysiphe convolvuli *(n.d.– no data)

**N**^**o**^	**Peptide**	**Sequence frequency**	**Percentage among all sequences**	**Common Name of Target**	**Length (aa)**	**Molecular Weight**	**Coverage [%]**	**Characterized in**	**References**
1.	KHJ33942.1	151	11.65	putative ubiquitin-40s ribosomal protein s31 fusion protein	155	17 883	10	*E. necator*	[77]
2.	RKF64955.1	75	5.79	Nucleoside diphosphate kinase	195	21 818	23	*E. neolycopersici* [*O. neolycopersici*]	[79]
3.	RKF61509.1	62	4.78	hypothetical protein OnM2_041055	200	22 667	15	*E. neolycopersici*	[79]
4.	RKF58703.1	60	4.63	Heat shock 70 kDa protein	647	70 592	5	*E. neolycopersici*	[79]
5.	KHJ31179.1	51	3.94	putative glycerol dehydrogenase	313	35 102	3	*E. necator*	[77]
6.	KHJ32358.1	42	3.24	putative heat shock protein 90	702	79 778	9	*E. necator*	[77]
7.	POS85160.1	40	3.09	putative malate dehydrogenase	331	34 479	3	*E. pulchra*	[76]
8.	EPQ67014.1	26	2.01	Enolase	438	47 161	15	*B. graminis* f. sp. *tritici* 96224	[78]
9.	KHJ33574.1	24	1.85	putative rho gdp-dissociation inhibitor	200	22 723	5	*E. necator*	[77]
10.	KHJ34285.1	23	1.77	putative neuronal calcium sensor 1	190	22 021	5	*E. necator*	[77]
11.	KHJ31784.1	23	1.77	putative nadp-dependent leukotriene b4 12-hydroxydehydrogenase	353	38 636	6	*E. necator*	[77]
12.	TQS35364.1	22	1.7	hypothetical protein Golomagni_04217	741	85 308	3	*G. magnicellulatus*	[20]
13.	POS82497.1	21	1.62	hypothetical protein EPUL_006059	116	13 254	18	*E. pulchra*	[76]
14.	KHJ34935.1	21	1.62	putative actin-bundling protein sac6	651	73 806	1	*E. necator*	[77]
15.	EPQ67394.1	20	1.54	Protein component of the small (40S) ribosomal subunit	285	31 351	13	*B. graminis* f. sp. *tritici* 96224	[78]
16.	KHJ30593.1	19	1.47	putative calcineurin regulatory subunit b	190	21 357	6	*E. necator*	[77]
17.	KHJ34217.1	17	1.31	putative pyridoxamine 5 -phosphate oxidase	248	28 922	4	*E. necator*	[77]
18.	POS82848.1	17	1.31	NAD(P)-binding protein	292	31 492	4	*E. pulchra*	[76]
19.	POS85004.1	15	1.16	putative fumarate reductase protein	601	65 133	6	*E. pulchra*	[76]

**Fig. 2:**
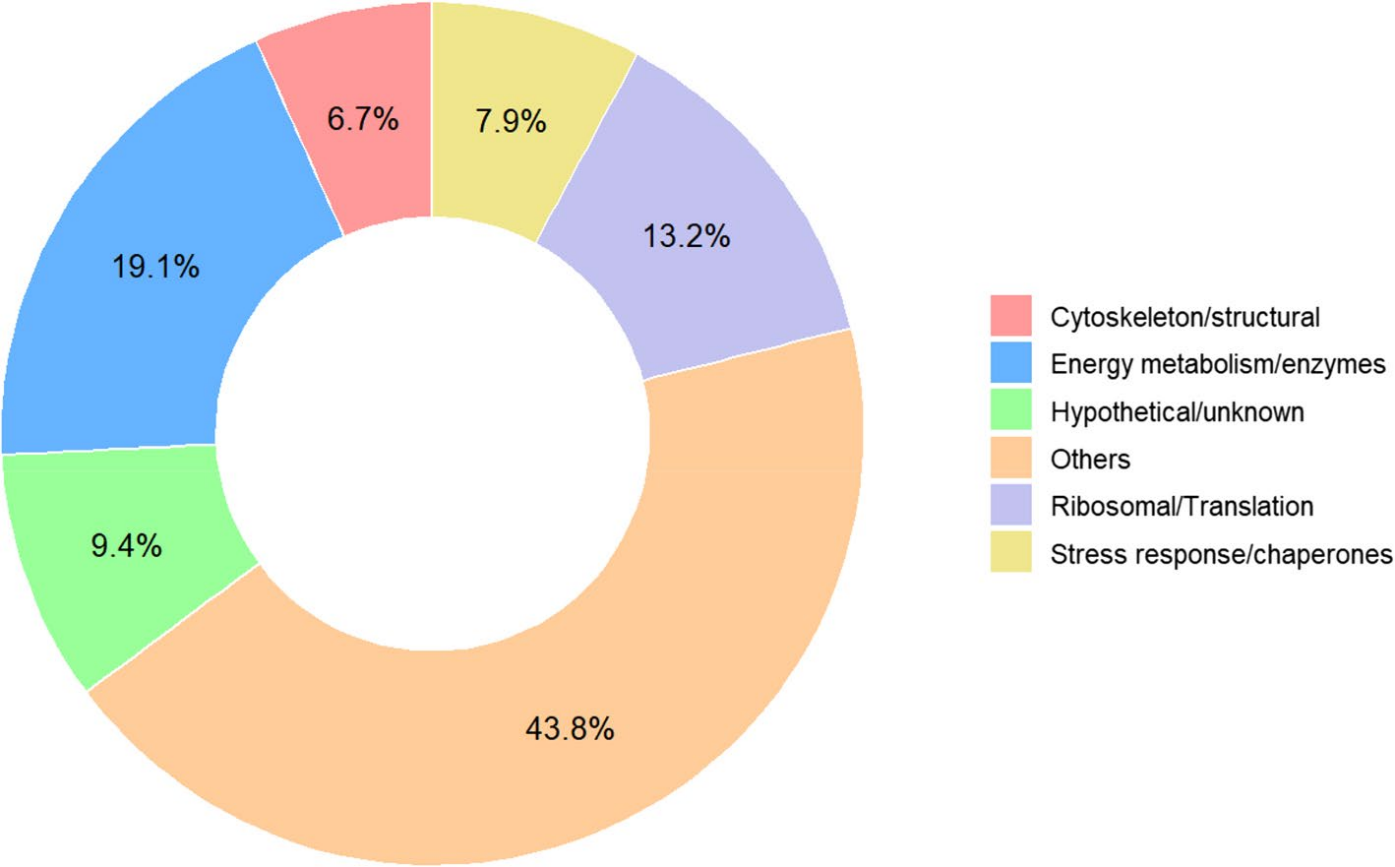
Functional classification of the most abundant proteins identified in *E. convolvuli*. The donut chart illustrates the relative abundance of proteins, grouped by their predicted or known biological functions. Percentages represent the proportion of each functional category among all identified sequences

The functional classification of the most abundant proteins in E. convolvuli, shown in Fig. 2, reveals that the largest proportion (43.8%) falls under the category “Other,” which may indicate the presence of unique metabolic or structural functions specific to this species. This is followed by enzymes involved in energy metabolism (19.1%) and ribosomal/translational proteins (13.2%), suggesting active protein synthesis and metabolic processes. Additionally, proteins of unknown function (9.4%) and stress response-related proteins/chaperones (7.9%) also contribute significantly. A smaller fraction is represented by cytoskeletal and structural proteins (6.7%). This distribution suggests a potential adaptation of the pathogen to environmental conditions and host interaction.

Among the most abundantly produced proteins in *E. convolvuli*, the ribosomal protein S31 (KHJ33942.1) was identified, which was also present in *E. palczewskii*. This protein is found in other species and was initially detected in *E. necator*. The protein identification was performed relying on its homology with proteins from other species.

Among the abundant proteins in *E. convolvuli*, several HSP were identified. The Hsp70 kDa protein (RKF58703.1) is the most frequent, accounting for 4.63% of all peptides. This protein, also characterized in *E. palczewskii*, is present in *E. necator* and *Oidium neolycopersici*, with 647 amino acids and a mass of 70.592 kDa [14–16]. Another essential HSP is Hsp90 kDa (KHJ32358.1), with a sequence comparable to peptides found in *O. neolycopersici* and *E. necator* [17].

Nucleoside diphosphate kinase (NDPK, RKF64955.1), constituting 5.79% of the peptides, belongs to the adenylate kinase group. These enzymes are commonly found in both prokaryotic and eukaryotic organisms. The peptide consists of 195 amino acids with a molecular mass of 21.818 kDa and has been previously characterized in *E. neolycopersici* [18].

Glycerol dehydrogenase (KHJ31179.1) accounts for 3.94% of all peptide sequences. It is a protein of 313 amino acids and has a mass of 35.102 kDa. It was also detected in *E*. *necator*. Glycerol dehydrogenase catalyzes the oxidation of glycerol to glycerol-3-phosphate, a key process in glycerol metabolism. Malate dehydrogenase (MDH, POS85160.1) constitutes 3.09% of all protein sequences. It has a length of 331 amino acids and a mass of 34.479 kDa. In *Erysiphe pulchra*, MDH also catalyzes the reversible conversion of malate to oxaloacetate, which is essential in the citric acid cycle and energy production [19, 20].

The Rho GDP dissociation inhibitor (GDI, KHJ33574.1) is a protein likely to be a Rho GDP inhibitor composed of 200 amino acids with a mass of 22.723 kDa, constituting 1.85% of the proteome. GDP dissociation inhibitors regulate the reactions of conversion of GTP to GDP [21].

Neuronal calcium sensor-1 (NCS-1, KHJ34285.1) in *E*. *convolvuli* constitutes 1.77% of all peptide sequences and comprises 190 amino acids with a mass of 22.021 kDa. NCS-1 participates in calcium signaling, which plays a role in various processes, including pathogenesis in fungi [22].

The hypothetical protein Golomagni_04217 (TQS35364.1) comprises 741 amino acids with a mass of 85.308 kDa, similar to a peptide sequence identified in *Golovinomyces magnicellulatus*. The functions of this protein are currently unknown. Another hypothetical protein, EPUL_006059 (POS82497.1), comprises 116 amino acids with a mass of 13.254 kDa, constituting 1.62% of all proteins. The function of this protein is unknown [23, 24].

Actin-binding protein sac6 (KHJ34935.1) comprises 651 amino acids with a mass of 73.806 kDa. The protein of the small (40 S) ribosomal subunit (EPQ67394.1) comprises 285 amino acids with a mass of 31.351 kDa, constituting 1.54% of the proteome [25].

Calcineurin B subunit (protein phosphatase 2B, KHJ30593.1) comprises 190 amino acids and has a mass of 21.357 kDa. Calcineurin regulates biological processes by dephosphorylating serine and threonine residues in proteins, crucial for fungal adaptation to environmental changes [26].

Pyridoxamine 5’-phosphate oxidase (PNP, KHJ34217.1) comprises 248 amino acids with a mass of 28.922 kDa. This enzyme is essential for converting PMP to the biologically active vitamin B6 (PLP) form [27].

NAD(P)-binding protein (POS82848.1) comprises 292 amino acids with a mass of 31.492 kDa [28]. Fumarate reductase (POS85004.1) consists of 601 amino acids with a mass of 65.133 kDa [29].

Endo-1,3-beta-glucanase (RKF61548.1) comprises 869 amino acids and has a mass of 95.709 kDa. This enzyme breaks down 1,3-beta-glucans, which are crucial for cell wall remodeling and defensive responses [30].

6-phosphogluconate dehydrogenase (6PGDH, KHJ30745.1) is a peptide composed of 493 amino acids with a mass of 54.687 kDa. The calmodulin-domain protein (KHJ32187.1) is a protein of about 28 kDa, composed of 244 amino acids. Calmodulin-domain proteins are multifunctional and are involved in key metabolic and developmental processes, such as lipid metabolism, signaling, reproduction, and pathogenesis [31].

6-phosphogluconate (RKF54735.1) is a peptide composed of 493 amino acids with a mass of 54.570 kDa. The pentose phosphate pathway converts it into ribulose-5-phosphate and CO₂, contributing to the production of pentoses and NADPH [31].

The hypothetical protein with an unknown function, Golomagni_05682 (TQS33957.1), is a 185-amino-acid peptide with a mass of 20.577 kDa, also detected in *G. magnicellulatus*. The NADP-binding protein (POS85221.1) is a 358-amino-acid peptide with a mass of 38.956 kDa [17, 32].

In the proteomic analysis, several proteins were identified in *E. convolvuli* that are absent in *E. palczewskii*. These unique proteins could provide insights into the distinct biological and pathogenic characteristics of *E. convolvuli* (Table 4). Among these proteins, the most notable include:

- NAD(P)-binding protein (POS82848.1): with a length of 292 amino acids and a molecular weight of 31,492 Da, this protein shows a 4% coverage and has been characterized in *E. pulchra*. NAD(P)-binding proteins are involved in redox reactions, which are crucial for metabolic processes [33]. Although NAD(P)-binding proteins serve as an essential cofactor in numerous metabolic activities, and proteins that bind to it are commonly found, the notion of an allergy specifically targeting these proteins is not recognized in scientific literature. However, proteins that bind to NAD(P), especially when altered or introduced uniquely, have the potential to be immunogenic, meaning they can trigger an immune response by exposing new epitopes or hiding existing ones [34].
- Endo-1,3-beta-glucanase (RKF61548.1): this large protein, consisting of 869 amino acids and having a molecular weight of 95,709 Da, has a 1% coverage and has been characterized in *O. neolycopersici*. Endo-1,3-beta-glucanases are enzymes that degrade glucans, playing a role in cell wall remodeling and pathogenesis [30, 35]. While endo-1,3-beta-glucanase itself may not be a major allergen, some studies suggest it can be part of the allergenic profile of certain plants like olive or associated with allergies to other allergens, such as pollen [36]. The immunogenic characteristics of endo-1,3-beta-glucanases can vary based on their origin and application. For instance, certain endo-1,3-beta-glucanases, such as those derived from *Chlamys albidus* scallops, can activate the immune system. Additionally, these enzymes can be utilized to boost the immunogenicity of β-glucans themselves [37].
- 6-phosphogluconate dehydrogenase (KHJ30745.1): this enzyme, with a length of 493 amino acids and a molecular weight of 54,687 Da, shows a 4% coverage and has been characterized in *E. necator*. It participates in the pentose phosphate pathway, a crucial metabolic pathway for cellular processes [13]. 6-phosphogluconate dehydrogenase (6PGD) has been shown to play a role in modulating CD8 + T cell activation and differentiation. Additionally, 6PGD is involved in metabolic reprogramming and has been implicated in cancer and infectious diseases [38].
- Putative caleosin domain-containing protein (KHJ32187.1): this protein has 244 amino acids, a molecular weight of 28,003 Da, and a 6% coverage. It has also been characterized in *E. necator*. Caleosins are associated with lipid bodies and are involved in stress responses and pathogen interactions [39]. The involvement of caleosins in stress reactions and defense against pathogens suggests they might possess immunogenic characteristics. When organisms encounter stressors or pathogens, caleosins or their fragments may be released, potentially triggering a plant immune response [40].
- RNP domain-containing protein (KHJ31756.1): this protein comprises 466 amino acids with a molecular weight of 50,419 Da and a 3% coverage, it has been characterized in *E. necator*. RNP domain-containing proteins are involved in RNA processing and regulation [41]. Its role in inflammatory processes and allergy is not known.
- 6-phosphogluconate dehydrogenase, decarboxylating 2 (RKF54735.1): this enzyme, similar to the previously mentioned dehydrogenase, has 493 amino acids and a molecular weight of 54,570 Da with a 4% coverage; it has been characterized in *Golovinomyces cichoracearum* [13].
- Hypothetical protein Golomagni_05682 (TQS33957.1): this minor protein, consisting of 185 amino acids and a molecular weight of 20,577 Da with a 3% coverage, has been characterized in *G. magnicellulatus* [42]. Its function remains unknown.
- Putative family transcriptional regulator protein (RKF59446.1): with 163 amino acids, a molecular weight of 18,814 Da, and a 9% coverage, this protein has been characterized in *O. neolycopersici*. Transcriptional regulators control gene expression, influencing various cellular processes [43]. The term “putative family transcriptional regulator protein inflammatory reaction” describes proteins believed to control the expression of genes associated with inflammation. Although these proteins have not been fully characterized, their function indicates a potential role in the inflammatory process [44].
- NAD(P)-binding protein (POS85221.1): this NAD(P)-binding protein has 358 amino acids, a molecular weight of 38,956 Da, and a 4% coverage. It has been characterized in *E. pulchra* [33].
- Glycoside hydrolase family 38 protein (KHJ30493.1): this large enzyme, with 1,077 amino acids and a molecular weight of 124,060 Da with a 1% coverage, has been characterized in *E. necator*. Glycoside hydrolases are involved in breaking down complex carbohydrates [45]. There are no available references concerning its role in inflammatory responses.

#### Comparative interpretation of clustered sequence similarity and expression-level heatmaps

**Fig. 3:**
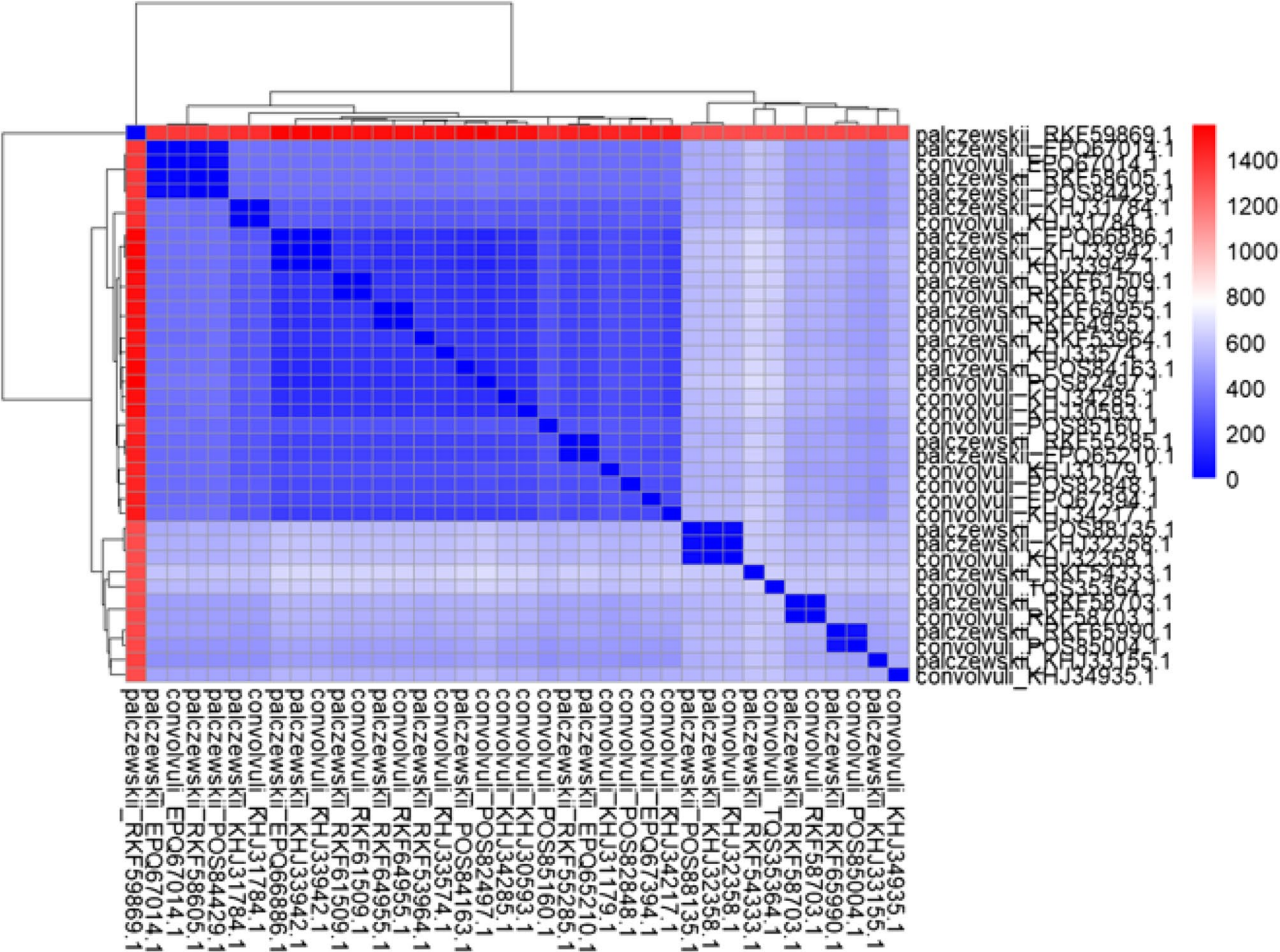
A clustered heatmap displays the distance matrix between the protein sequences of two strains: *E. palczewskii* and *E. convolvuli*. The dendrogram was generated based on pairwise comparisons of peptide FASTA sequences. Color intensity reflects similarity values, ranging from lowest (dark blue) to highest (red)

**Fig. 4:**
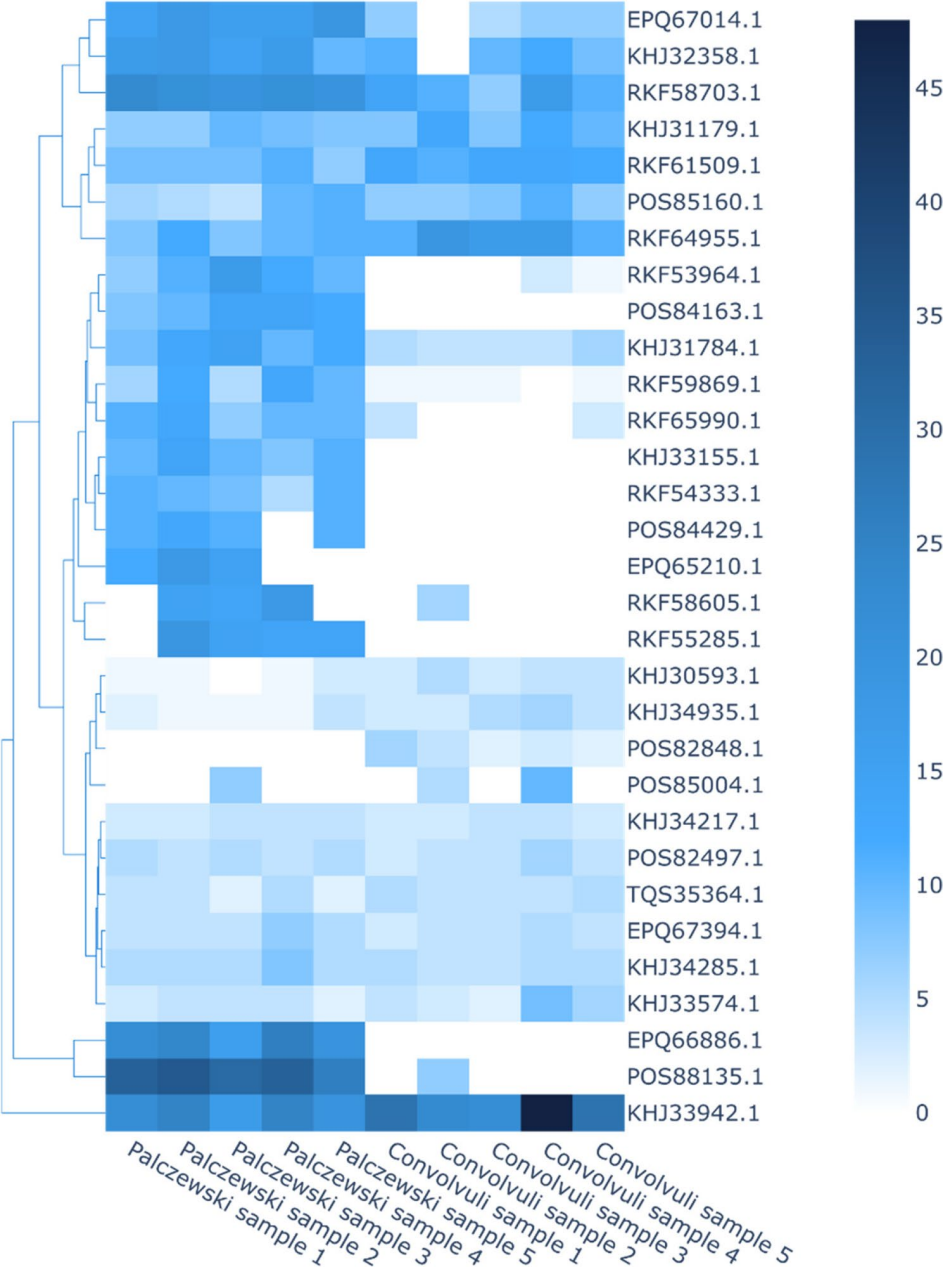
Heatmap displaying the relative abundance of proteins identified in *E. palczewskii* and *E. convolvuli* across five biological replicates per species. Color intensity indicates normalized expression levels

Two complementary heatmaps (Figs. 3 and 4) were generated to investigate the structural and functional relationships among proteins identified in *E. palczewskii* and *E. convolvuli*.

Figure 3 presents a clustered heatmap of pairwise protein sequence similarity between the two fungal species. Clustering was conducted based on global alignments of protein sequences, enabling visualization of conserved and divergent protein groups. The dendrogram illustrates a clear grouping of homologous proteins, with a prominent cluster of highly similar sequences (represented in red), indicating the presence of conserved core proteins involved in fundamental cellular processes. In contrast, several distinct branches of low-similarity pairs (shown in blue) may correspond to species-specific proteins, potentially linked to unique metabolic pathways or host-specific adaptations.

Figure 4 presents a heatmap of relative protein abundance derived from LC-MS/MS quantification across five biological replicates for each species. This representation illustrates differences in expression levels among shared and unique proteins. While both species express a common set of highly abundant proteins, *E. palczewskii* displays elevated levels of proteins involved in secondary metabolite biosynthesis. In contrast, *E. convolvuli* shows greater abundance of proteins related to oxidative stress response and detoxification. These expression patterns suggest functional divergence influenced by ecological pressures and distinct host interactions.

Together, the sequence-based clustering and expression-level profiles offer integrated insights into the evolutionary conservation and functional specialization of proteins in these two phytopathogenic microfungi.

**Table 4:** Most unique proteins identified in *Erysiphe convolvuli*

**N**^**o**^	**Peptide**	**Common Name of Target**	**LENGTH (aa)**	**Molecular Weight (da)**	**Coverage [%]**	**Characterized in**	**References**
1.	3:POS82848.1	NAD(P)-binding protein	292	31 492	4	*E. pulchra*	[76]
2.	3::RKF61548.1	putative endo-1,3-beta-glucanase	869	95 709	1	*O. neolycopersici*	[79]
3.	2::KHJ30745.1	putative 6-phosphogluconate dehydrogenase	493	54 687	4	*E. necator*	[77]
4.	2::KHJ32187.1	putative caleosin domain containing protein	244	28 003	6	*E. necator*	[77]
5.	3::KHJ31756.1	putative rnp domain-containing protein	466	50 419	3	*E. necator*	[77]
6.	2::RKF54735.1	6-phosphogluconate dehydrogenase, decarboxylating 2	493	54 570	4	*G. cichoracearum*	[79]
7.	3::TQS33957.1	hypothetical protein Golomagni_05682	185	20 577	3	*G. magnicellulatus*	[20]
8.	3::RKF59446.1	putative family transcriptional regulator protein	163	18 814	9	*O. neolycopersici*	[79]
9.	3::POS85221.1	NAD(P)-binding protein	358	38 956	4	*E. pulchra*	[76]
10.	2::KHJ30493.1	putative glycoside hydrolase family 38 protein	1 077	124 060	1	*E. necator*	[77]

### Analysis of fatty acid composition

**Fig. 5:**
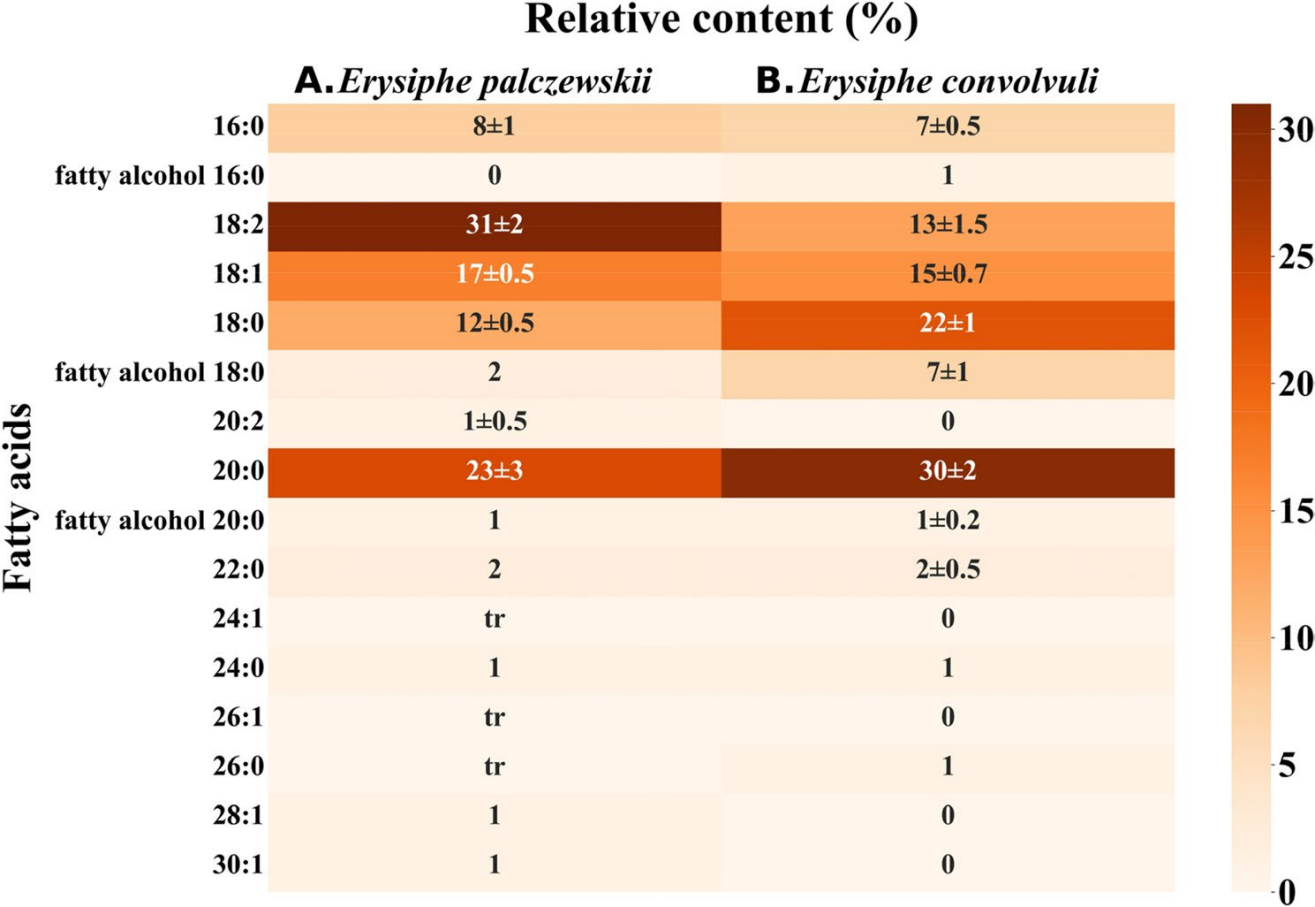
Fatty acid composition and relative content (%) in *E. palczewskii* (**A**) *and E. convolvuli* (**B**) spores

The fatty acid (FA) analysis in methyl esters showed that approx. half of all FAs in the *E. palczewskii* spores were unsaturated. Among them, 18:2 (31 ± 2%) and 18:1 (17 ± 0.5%) FAs dominated. This fungus also synthesized small amounts of unsaturated FAs with long chains: 28:1 and 30:1. Among saturated acids, 20:0 (23 ± 3%) and 18:0 (12 ± 0.5%) FAs dominated. The *E. palczewskii* spores also contained fatty alcohols (18:0, 20:0) (Fig. 5A). In the *E. convolvuli* spores, FAs with a chain length from 16 to 26 carbon atoms were synthesized. They produced both saturated acids and mono- and di-unsaturated acids. The saturated acids were dominated by 20:0, 18:0, and 16:0, constituting approx. 60% of all FAs. Substantial content of unsaturated FAs was also identified, among which 18:1 (15 ± 0.7%) and 18:2 (13 ± 1.5%) were the most abundant. A characteristic feature of the *E. convolvuli* spores was the presence of three fatty alcohols, of which the 18-carbon alcohol constituted 7 ± 1% (Fig. 5B).

The analysis of the composition of FAs contained in the cells of the hosts of the tested fungi (*C arborescens* for *E. palczewskii* and *C. arvensis* L for *E. convolvuli*) showed a different composition than the FA profile of the fungi, confirming the purity of the collected fungal spores (Table S1, Table S2).

### Analysis of the carbohydrate composition

**Fig. 6:**
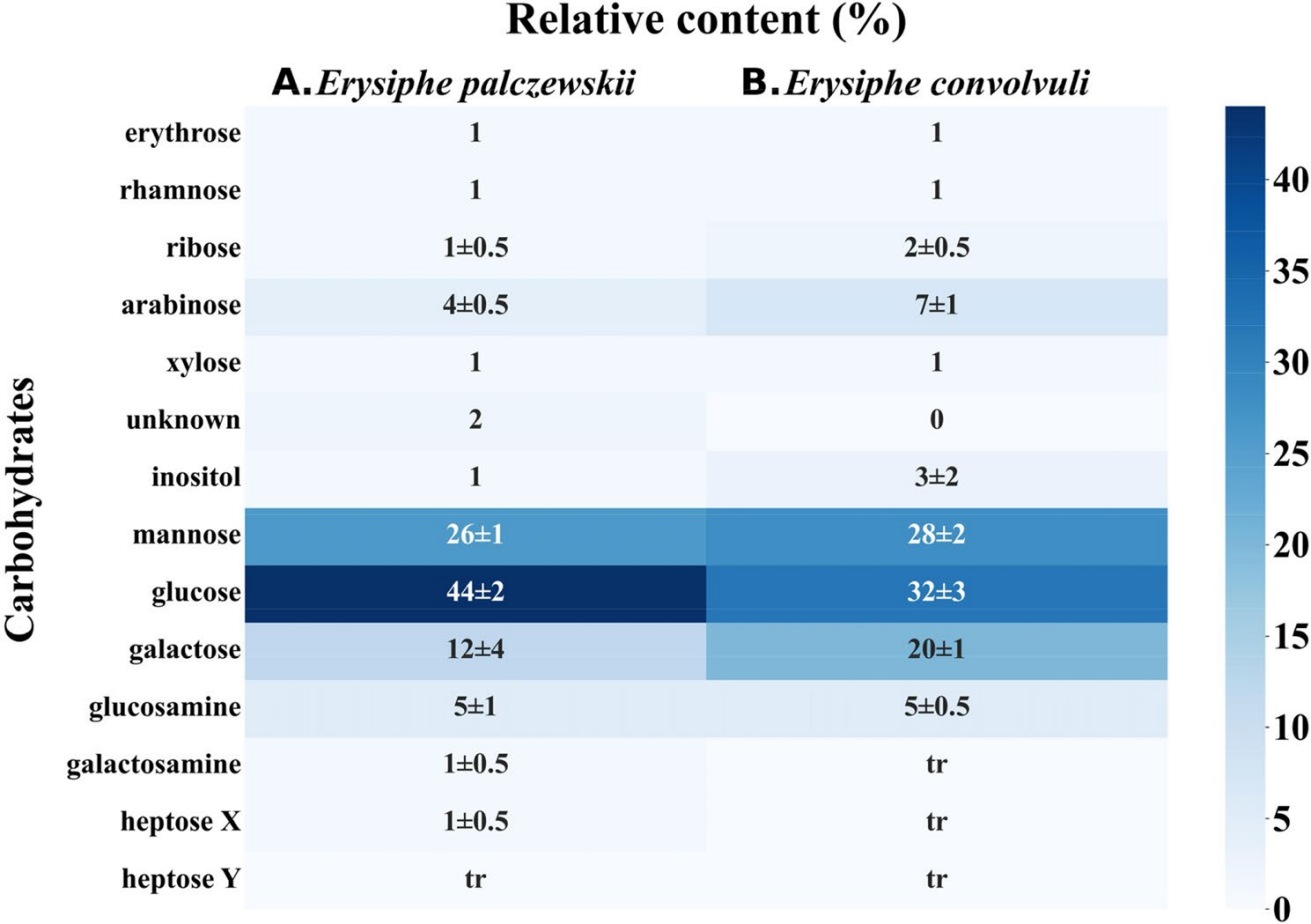
Carbohydrate composition and relative content (%) in *E. palczewskii* (**A**) and *E. convolvuli* (**B**) spores (tr– trace amounts, less than 0.5%)

The *E. palczewskii* spores were characterized by a diverse carbohydrate composition. The dominant sugars were hexoses, including glucose, mannose, and galactose, which constituted approx. 82% of all sugars in the *E. palczewskii* spores. In addition, pentoses (arabinose − 4 ± 0.5%; ribose − 1 ± 0.5%; xylose − 1%) and hexosamines (glucosamine − 5 ± 1%; galactosamine − 1 ± 0.5%) were found. The relative content of erythrose, rhamnose, xylose, inositol, and heptoses did not exceed 1% (Fig. 6A). A substantial amount of hexoses (glucose − 32 ± 3%, mannose − 28 ± 2%, and galactose − 20 ± 1%,) was identified in the spores of *E. convolvuli*. In turn, the content of pentoses was approx. 10%. The carbohydrate composition of this species was characterized by the presence of amino sugars, with glucosamine being the most abundant (5 ± 0.5%). Similar to *E. palczewskii*, the spores of *E. convolvuli* also contained erythrose and rhamnose, although their content remained below 1%. Additionally, trace amounts of heptoses were detected (Fig. 6B).

## Discussion

Inhalant allergens are essential triggers of respiratory allergies [46]. Fungal spores are ubiquitous airborne biological particles recognized as one of the most critical inhalant allergens since the 12th century [47]. Although the incidence of fungal allergy is not precisely known, it is estimated to range from 3 to 10% in the general population (depending on the climatic conditions of a given area) [48]. The best documented allergenic properties have been reported for common species of the genera *Cladosporium*,* Penicillium*,* Aspergillus*, and *Alternaria* [49]. However, the panel of fungal species with potential allergenic properties is still expanding. Our recent work has shown that two species of the genus *Erysiphe*, *E. palczewskii* and *E. convolvuli*, have proallergenic activity [5]. These species have not been characterized biochemically so far. Therefore, in this work, we present the characterization of proteins, FAs, and sugars, considering that proteins or their combinations with lipids are the most common allergens.

Recent studies have begun to explore the potential allergenic effects of these pathogens. Sztandera-Tymoczek et al. (2023) demonstrated in vitro that E. *palczewskii* and E. *convolvuli* exhibit significant proallergenic activity, highlighting their potential to trigger allergic responses in sensitive individuals [5]. This research emphasizes the need for further investigation into the molecular characteristics of these fungi to understand the specific allergens they produce and how these may interact with the human immune system. Such findings are essential, as the identification of potential allergens can inform public health measures and guide the development of diagnostic tools for identifying fungal-induced allergies.

Furthermore, the biochemical composition of these fungi has been analyzed in terms of their allergenic potential. Studies by Al-Obaidi et al. (2021) explored the broader context of fungal allergens and their role in human health, suggesting that fungi, including those of the Erysiphe genus, could serve as potential sources of immunogenic compounds that contribute to allergic diseases [50]. The findings from Cunha et al. (2023) also provide insights into the broader implications of fungal metabolites in allergic and inflammatory conditions. While their focus was on macrofungi, their work offers valuable parallels to the potential health risks associated with microfungi like E. *palczewskii* and E. *convolvuli* [51].

Additionally, Al-Obaidi et al. (2021) have discussed the broader applications of fungal-derived nutraceuticals and mycopharmaceuticals, underscoring the growing recognition of fungi as allergens and potential sources of beneficial compounds. Understanding the dual role of these organisms as both allergens and sources of bioactive compounds could be instrumental in developing strategies to mitigate fungal-related allergic reactions while harnessing their therapeutic potential [52].

Characterizing the protein profiles of *E. palczewskii* and *E. convolvuli* may have implications for plant and human health protection. For example, fungal pathogens, such as *Fusarium graminearum*, have been the subject of extensive proteomic studies carried out to identify proteins involved in pathogenicity and host resistance. Similarly, the extracellular proteome of *Penicillium chrysogenum* has been analyzed, highlighting the importance of this microorganism as a significant indoor allergen [53].

The comparative proteomic analysis of *E. convolvuli* and *E. palczewskii* provides significant insights into the unique and shared protein profiles of these two powdery mildew fungi. A total of 770 protein sequences were identified in *E. convolvuli*, with 47% (362 sequences) being unique to this species, underscoring the genetic and functional diversity within the genus *Erysiphe*. The characterization of shared sequences, with 359 peptides common to *E. convolvuli* and *E. palczewskii*, highlights the evolutionary and functional conservation between these fungi.

The discovery that 362 protein sequences are unique to *E*. *convolvuli* suggests species-specific adaptations that could be linked to its pathogenicity, environmental interactions, and survival strategies. The absence of these sequences in *E*. *palczewskii* implies distinct biological roles that may contribute to the differences in host specificity, virulence, and ecological niches occupied by these two species.

The presence of 359 shared protein sequences between *E*. *convolvuli* and *E*. *palczewskii* indicates a substantial overlap in their core proteomes. These shared proteins likely represent fundamental biological processes essential for the survival and growth of both fungi. The conserved nature of these sequences across different species within the genus *Erysiphe* suggests that they play critical roles in essential cellular functions, such as metabolism, protein synthesis, and stress response.

The proteomic analysis revealed several highly abundant proteins in *E*. *convolvuli*, many of which are also present in *E*. *palczewskii* and other related fungi. For instance, the ribosomal protein s31 (KHJ33942.1) and the HSP 70 kDa protein (RKF58703.1) are not only abundant in both *E*. *convolvuli* and *E*. *palczewskii* but are also found in *E. necator* and *O. neolycopersici*. The widespread presence of these proteins across multiple species suggests their essential role in maintaining cellular integrity and responding to environmental stress.

Proteins such as HSP70 and HSP90 are involved in essential cellular functions like protein folding and preventing protein clumping, which are vital for the survival of fungi under stressful conditions. Although HSPs play a role in the stress response of fungi, including those that trigger allergic reactions, they probably do not directly cause the allergic reaction itself [54]. However, one example of fungal allergen is Aspf 12, a HSP90 protein derived from Aspergillus, which might be involved in the organism’s stress response [55]. Additionally, HSP90α plays a role in the cGAS-STING-ER stress pathway associated with HDM-induced asthma, resulting in the pyroptosis of airway epithelial cells [56]. The identification of HSP70 and HSP90 as highly abundant in both *E*. *convolvuli* and *E*. *palczewskii* underscores the importance of stress response mechanisms in these fungi, participating thereby in allergic response.

The presence of key metabolic enzymes, such as glycerol dehydrogenase, malate dehydrogenase, and nucleoside diphosphate kinase (NDPK), in the proteomes of both species indicates their involvement in essential metabolic pathways. These enzymes are critical for energy production, carbon metabolism, and nucleotide synthesis, which are fundamental for the growth and reproduction of fungi, as well as play important role in the adaptation of fungi to various microenvironments [57]. The comparative abundance of these enzymes in.

*E. convolvuli* and *E. palczewskii* suggest similar metabolic strategies, although specific differences may reflect adaptations to different host plants or environmental conditions.

The characterization of hypothetical proteins, some of which are highly abundant, represents an exciting direction for future research. With unknown functions, these proteins may hold the key to novel biological processes and pathogenic mechanisms unique to these fungi. Further studies involving functional genomics and proteomics can shed light on the roles of these hypothetical proteins, providing new targets for controlling powdery mildew diseases.

Allergic sensitization is a multifactorial process. The allergenic activity of protein allergens is determined by their structural and physicochemical properties and innate immunomodulatory characteristics and their ability to bind hydrophobic ligands, such as lipids [58, 59]. These lipids may either interact directly as ligands with the allergen or be naturally present within the allergen source [60]. The qualitative and quantitative composition of FAs contained in different classes of lipids in all organisms is characteristic for each genus and species and depends on environmental conditions. Nevertheless, lipidomic analyses of fungi are rarely performed, in contrast to other groups of organisms [61]. The GLC/MS analysis results showed that the common plant pathogens studied in this work synthesize saturated and unsaturated FAs with acyl residue lengths from 14 to 30 carbon atoms in the molecule. Among the identified FAs, 16:0, 18:0, 18:2, and 20:0 acids were common to the spores of both *Erysiphe* species. Gas-liquid chromatography results of six *Aspergillus* species (*A. caelatus*, *A. parasiticus*, *A. fumigatus*, *A. nomius*, *A. ochraceus*, and *A. flavus*) showed that they all contained 16:0, 18:0, 18:1, and 18:2 FAs, constituting about 95% of the total FA content [62]. Mahmoud et al. analyzed the FA profiles of five *Penicillium* species (*P. chrysogenum*,* P. funiculosum*,* P. griseofulvum*,* P. implicatum*, and *P. oxalicum*). The number of carbon atoms in the chain ranged from 14 to 20, and the most frequently extracted FAs were 18:1 and 18:2, which accounted for 80% or more of the total FAs [63]. Other compounds, including carbohydrates and lipids, may accompany many allergenic proteins recognized by IgE. When bound to allergenic proteins, lipids form complexes that can be recognized by the innate immune system. Direct binding of lipids (FAs, glycolipids, or phospholipids) by allergenic proteins is possible due to their hydrophobic cavities or ionic bonds, resulting in protein-lipid complexes. This results in structural and biochemical changes in the protein, which modifies the Th2 response, but the mechanisms underlying these observations are largely unknown.

Furthermore, lipids occur together with proteins in allergenic substances. They are not the main target of the immune system, but they can influence the development of an allergic reaction by acting on dendritic cells, which in turn leads to the differentiation of Th2 cells recognizing peptides from allergenic proteins [64–66]. In this regard, the presence of the 18:2 FA in fungal species considered allergenic is worth noting. Linoleic acid is a polyunsaturated 18:2, and a metabolic precursor of arachidonic acid (AA; 20:4) [67]. The AA pathway is crucial in many inflammatory diseases, such as asthma. Linoleic acid is metabolized by lipoxygenases (LOXs) and cyclooxygenases (COXs), forming biologically active mediators– leukotrienes and prostaglandins, respectively [68]. They are strong mediators of inflammation, and their receptors are located on mast cells, eosinophils, B lymphocytes, and macrophages. They mediate bronchoconstriction, increased vascular permeability, eosinophil activation, and increased mucus secretion [69–71].

The slight differences between the individual profiles indicate a similar carbohydrate composition of *E. convolvuli* and *E. palczewskii.* The identified carbohydrates include hexoses, with predominant glucose, and pentoses, with a significant percentage of arabinose. Another characteristic feature is the presence of amino sugars, such as glucosamine and/or galactosamine. Reports on identifying carbohydrates in fungi commonly considered allergenic are scarce and mostly date back to the 20th century. However, the identification of carbohydrates in the cell wall of *A. fumigatus* showed that glucose represented almost 59% of these compounds, while galactose and mannose accounted for approximately 23% and 11%, respectively. The cell wall of this fungal species was also characterized by the presence of slightly over 7% of amino sugars (N-acetylglucosamine and N-acetylgalactosamine) [72]. It is known that the fungal cell wall contains chitin, a polysaccharide consisting of β−1,4-N-acetylglucosamine, which is linked to β-glucans, galactomannans, and mannoproteins, thus forming the structural foundation of the cell wall [73]. Studies conducted by Reese et al. confirmed the ability of chitin to initiate innate immune responses associated with allergy and asthma. They noted that administration of chitin to mice induced the recruitment of IL-4-expressing innate immune cells, including eosinophils and basophils, to the lungs and/or peritoneum [74]. Furthermore, chitin increased the total and antigen-specific IgE levels in the serum of mice immunized with the *A. fumigatus* antigen [75].

## Conclusion

Our biochemical analyses of *E. convolvuli* and *E. palczewskii* extracts revealed the allergenic potential of these fungi, highlighting the role of proteins, lipids, and carbohydrates as triggers of respiratory allergic responses.

Our study expands our understanding of the biology of these microfungi by identifying proteins such as heat shock proteins (HSPs) and specific enzymes involved in fungal metabolism and stress response. Additionally, the discovery of proteins present in one species but absent in the other suggests potential differences in their pathogenicity and allergenicity mechanisms.

The results indicate that these proteins may be responsible for immune reactivity variation and their interactions with respiratory epithelial cells. However, protein identification remains somewhat certain, particularly for proteins recognized based on homology with proteins from other *Erysiphales* species, which may result in less precise outcomes.

These findings emphasize the significance of ongoing research into the allergenic properties of fungi such as E. *palczewskii* and E. *convolvuli*. The growing body of knowledge on fungal allergens will improve our understanding of allergic diseases and may lead to new allergy prevention and treatment strategies.

Future studies should focus on identifying specific allergenic proteins and elucidating their interactions with respiratory epithelial cells to develop targeted therapeutic strategies for managing fungus-induced allergic conditions.

## Methods

### Sample collection and Preparation for analysis

The fungi chosen for this study are biotrophic organisms, i.e. they cannot be cultivated on artificial media in a laboratory setting. As a result, the research material was sourced from the natural environment. Specimens of plant organs infected by powdery mildew (*Erysiphales*) species were collected in Lublin: *E. palczewskii* on *C. arborescens* on August 31, 2021 (LBL M‒033118) and *E. convolvuli* on *C. arvensis* on August 10, 2021 and between September 1–10, 2021 (LBL M‒033119). The host plants are commonly found in parks and gardens (*C. arborescens*) and wasteland (*C. arvensis*); they are not protected species and do not occur in conservation areas.

The collected material consisting of leaves and infecting fungi was air-dried and preserved in the herbarium of Maria Curie-Skłodowska University in Lublin (LBL). Initially, the morphological structures of the specimens were examined through microscopic preparations stained with lactophenol cotton blue and observed under an Olympus BX53 light microscope at magnifications of 40x, 100x, 400x, and 600x. Microphotographs of diagnostic fungal structures were captured using an Olympus SC180 digital camera and an Olympus SZ10 stereoscopic microscope equipped with an XC50 camera. The samples were coated with gold using an Emitech K550X Sputter Coater and analyzed under a TESCAN Vega 3 LMU scanning electron microscope (SEM) for visualization. In addition to the microscopic identification of the species, genetic identification was also carried out, which confirmed that the collected biological material represented *E. palczewskii* and *E. convolvuli* [5].

Subsequently, samples containing fungal morphological structures, such as mycelium, chasmothecia (fruiting bodies), conidiophores, and conidia, were prepared for further laboratory analyses under an Olympus SZ61 stereoscopic microscope. The collected material was placed in test tubes, subjected to liquid nitrogen vapor for 24 h, and then pulverized using a mortar and pestle. The powdered fungal material was used to prepare crude extracts for further studies.

The crude fungal extracts prepared using the method described in our previous paper [5] were aliquoted into 100 µL portions and stored at −80 °C for further analysis.

## Biochemical characteristics of the microfungal extracts

### Biochemical identification of proteins in the fungal extracts

#### Mass spectrometry

##### Protein digestion

Proteomics analysis was performed at the Mass Spectrometry Laboratory at the Institute of Biochemistry and Biophysics PAS. Each sample (50 µg) was diluted to 50 µL with 100 mM ammonium bicarbonate buffer (ABC). Cysteine residues were reduced by incubating with 10 mM tris(2-carboxyethyl) phosphine (TCEP) for 1 hour at 60 °C, followed by a 10-minute incubation at room temperature with 30 mM methyl methanethiosulfonate (MMTS). Digestion was carried out overnight using trypsin (Promega) at an enzyme-to-protein ratio of 1:25 at 37 °C. After the digestion, the peptides were cleaned using OASIS HLB 10 mg columns (Waters) following the manufacturer’s protocol. Briefly, the cartridges were preconditioned with 1 mL methanol and washed with 1 mL of 0.1% formic acid. After loading the samples and rinsing with 1 mL of 0.1% formic acid, the peptides were eluted from the columns with 200 µL of 80% acetonitrile (ACN) and 0.1% formic acid. The eluates were dried in a SpeedVac and resuspended in 0.1% formic acid. Peptide concentrations were measured using the Pierce Quantitative Colorimetric Peptide Assay (Thermo Scientific).

##### Mass spectrometry measurements

The peptides were analyzed using an LC-MS system consisting of an Evosep One (Evosep Biosystems) coupled with an Orbitrap Exploris 480 mass spectrometer (Thermo Fisher Scientific). In accordance with the manufacturer’s instructions, 1 µg of peptides from each sample was loaded onto disposable Evotip C18 trap columns (Evosep Biosystems). The bound peptides were washed three times with 100 µL of solvent and then covered with 300 µL of solvent A (0.1% formic acid in water). Chromatographic separation was performed using an 88-minute gradient on an analytical column (ReproSil Saphir C18, 1.5 μm beads, 150 μm ID, 15 cm length, Evosep Biosystems) at a flow rate of 220 nL/min. Data acquisition in the positive ion mode was performed using a using the data-dependent acquisition (DDA) method with the following settings: MS1 scans were obtained at a resolution of 60,000 with a normalized AGC target of 300% and automatic maximum injection time, and a scan range from 300 to 1600 m/z. MS2 scans were performed at a resolution of 15,000 using a standard normalized AGC target and automatic maximum injection time. The top 40 precursor ions were selected for the MS/MS analysis within an isolation window of 1.6 m/z. A dynamic exclusion period of 20 s was applied, with a mass tolerance of ± 10 ppm and a precursor intensity threshold of 5e3. Fragmentation was carried out using higher-energy collisional dissociation (HCD) with a normalized collision energy of 30%. The source parameters included a spray voltage of 2.1 kV, a funnel RF level of 40, and a heated capillary temperature of 275 °C.

### Data analysis

The data obtained were pre-processed with Mascot Distiller (version 2.8, Matrixscience) and searched with the Mascot Server engine (version 2.8, Matrixscience) against *Erysiphales* proteins (74 360 sequences) derived from NCBI (version 20211212) supplemented with popular MS contaminants (Crap database, 115 sequences). The other parameters were as follows: enzyme– trypsin, missed cleavages − 1, fixed modifications– methylthio (C), variable modifications– oxidation (M). Each file was subjected to offline mass recalibration, with resulting fragment mass tolerance of 0.01 Da and parent mass tolerance ranging from 19 to 5 ppm. FDR was calculated and kept under 1% for each file based on the target/decoy strategy with a Mascot-generated reverse database.

The Mascot score was used as the primary indicator of identification confidence to ensure the reliability of protein identifications. A Mascot score greater than 35 was considered a statistically significant identification (i.e., with a p-value of less than 0.05). To further assess the confidence in protein identifications, the False Discovery Rate (FDR) was calculated for each dataset using the target-decoy strategy with a Mascot-generated reverse database. The FDR was maintained below 5% for each sample, ensuring an appropriate confidence level in the identifications.

The bioinformatic analysis of the extracted peptide sequences was carried out in multiple stages. First, the data exported from the Mascot Server was converted into an appropriate format for further analysis. Python software (version 3.10) was then developed using the PyCharm Community Edition to process the data. The study aimed to compare and identify proteins present across different fungal species. The generated software was run on the downloaded files, which included such information as protein names, accession numbers, molecular weights, and functions. The software was designed to handle.xlsx files and utilized libraries like NumPy and OpenXL.

Additionally, R (version 4.5.0, RStudio 2024.12.1) was used to generate some visualizations, including heatmaps with hierarchical clustering and donut charts. The pheatmap package facilitated the creation of heatmaps with dendrograms, while ggplot2 was utilized to produce donut charts.

### Biochemical identification of fatty acids in the fungal extracts

The biological material in the form of fungal dry mass (3 mg) was suspended in 500 µL of a 2 M butanol HCl solution and heated at 85 °C for 90 min. After cooling the samples to room temperature, 2.5 mL of a 10% aqueous potassium hydroxide solution was added and then incubated for 30 min at 65 °C. The contents of the tubes were mixed with 2.5 mL of 1 M phosphoric acid. Then, FAs were extracted by adding a mixture of hexane/water (1/1, v/v). After intensive mixing (vortex Labnet), the samples were centrifuged (5000 × g, 15 min, RT) and the lower organic phases were collected. The extraction of free FAs was repeated three times. All the organic phases obtained were combined and evaporated in a stream of nitrogen. Then, 350 µL of 20% methanol in acetone and 350 µL of 0.02 M trimethylsilyldiazomethane (TMSD, Sigma-Aldrich) in hexane were added to the tubes and mixed thoroughly. After 30 min incubation at room temperature, the samples were evaporated to dryness using a vacuum evaporator (Bűchi R-100). The experiment was performed in three independent replicates. The obtained FAs in the form of methyl esters were suspended in 50 µL chloroform and injected into the column of a gas-liquid chromatograph coupled to mass spectrometry (GC/MS) [61].

### Biochemical identification of carbohydrates in the fungal extracts

To release carbohydrates from the fungal dry mass (3 mg), the samples were subjected to 4-h hydrolysis in 1 mL of 2 M trifluoroacetic acid (TFA) at 100 °C. After evaporating the tube contents using a vacuum evaporator, N-acetylation was carried out by adding 280 µL of a mixture of pyridine/acetic anhydride/methanol (1/1/4, v/v/v). The samples were left overnight at room temperature and dried in a nitrogen stream the next day. Then, reduction (300 µL of 1 M ammonia/NaBD4, 24 h, RT) was carried out and the samples were incubated at 46 °C for 2 h. After acidification with a few drops of 99.9% acetic acid, the contents of the tubes were evaporated in a nitrogen stream. Then, 300 µL of 5% acetic acid in methanol were added to completely remove borates and evaporate to dryness in a nitrogen stream. The samples were suspended in 300 µL of 99.8% methanol and dried using a vacuum evaporator– this step was repeated three times. Peracetylation was performed by treating the samples with 150 µL of pyridine and 150 µL of acetic anhydride. After 20-min incubation at 100 °C, the content of the tubes was evaporated in a nitrogen stream and a chloroform/water mixture (1/3, v/v) was added. After centrifugation (5000 × g, 15 min, RT), the chloroform phases were passed through a column of anhydrous sodium sulfate and evaporated to dryness. The isolation of carbohydrates was performed in three independent experiments. Sugars in the form of alditol acetates were suspended in 100 µL of chloroform and subjected to gas-liquid chromatography coupled with mass spectrometry (GLC/MS).

### Gas-liquid chromatography coupled to mass spectrometry (GLC-MS)

FA methyl esters and monosaccharide alditol acetates were analyzed by GLC-MS using a gas chromatograph (Agilent Technologies, instrument 7890 A, Santa Clara, CA, USA) connected to an MSD 5975 C detector (Inert XL EI/Cl). The chromatograph had an HP-5ms (SLB-5ms) capillary column (30 m × 0.25 mm; Sigma-Aldrich, USA). A temperature gradient from 150 °C (5 min) to 310 °C was applied at a rate of 5 °C/min, and the final temperature was maintained for 10 min. Individual FAs were identified by comparing the retention times of FA methyl esters and their standards. They were also based on the m/z values of the corresponding ions in the mass spectra. Saturated and monounsaturated FAs were identified based on the presence of ions at 74 and 87 m/z, the molecular ion [M]^+^, and the ions [M-31]^+^, [M-32]^+^, [M-29]^+^, [M-43]^+^, and [M-116]^+^. The methyl esters of 2-hydroxy FAs were identified based on the basic ion [M-59]^+^, which is formed as a result of the cleavage between the first and second carbon in the alkyl chain and allows the determination of the location of the hydroxyl group.

The identification of sugar components was carried out based on the analysis of characteristic mass spectra of their alditol acetates obtained from aldoses fragmented to form ions of 146 m/z (and a secondary ion of 104 m/z) and 218 m/z (and secondary ions of 158 and 116 m/z). They contained an alditol acetate fragment, including the C1 terminus labeled with m/z deuterium during NaBD_4_ reduction of sugar residues after hydrolysis. In contrast, ions of 145 m/z (and a secondary ion of 103 m/z), 217 m/z (and secondary ions of 157 and 115 m/z), and 289 m/z (and a secondary ion of 187 m/z) corresponded to fragments containing the C6 terminus of hexitol. The relative content (%) of each FA and carbohydrate was calculated from the ratio of its peak area to the sum of the areas of all peaks. Qualitative and quantitative results of FA and sugar analysis were presented in the form of heatmaps generated in the Python 3 programming language [Seaborn (version 0.12.1) and Matplotlib (version 3.6.2) libraries].

## Supplementary Information

Supplementary Material 1.

## Data Availability

All data are contained within the manuscript and supporting information. Additionally, the mass spectrometry proteomics data have been deposited to the ProteomeXchange Consortium via the PRIDE partner repository, with dataset identifiers PXD058325 and 10.6019/PXD058325:Project Webpage: https://www.ebi.ac.uk/pride/archive/projects/PXD058325FTP Download: https://ftp.pride.ebi.ac.uk/pride/data/archive/2025/06/PXD058325.

## Abbreviations

NDPK: Nucleoside diphosphate kinase
HSP: Heat shock protein
MDH: Malate dehydrogenase
NCS-1: Neuronal calcium sensor-1
6PGDH: 6-phosphogluconate dehydrogenase
FA: Fatty acid
