# Supplementary material for: Biochemical characteristics of extracts from proallergenic microfungi Erysiphe Palczewskii and Erysiphe convolvuli
Source: BMC Genomics. 2025 Aug 18;26:754. doi: 10.1186/s12864-025-11862-w (PMC12363095; doi:10.1186/s12864-025-11862-w)
Supplement: Supplementary file 1 — Supplementary Material 1. [file 12864_2025_11862_MOESM1_ESM.docx]

**Supporting information**

**Table S1.** The main **fatty acid (%) in *Caragana arborescens Lam.***

| **Retention time** | **Fatty acid** | **Relative content (%)** |
| --- | --- | --- |
| 10.44 | 14:0 | 2±0.5 |
| 14.89 | 16:1 | 6±1 |
| 14.99 | 16:0 | 58±7 |
| 16.96 | 17:0 | 1 |
| 18.85 | 18:0 | 17±6 |
| 18.96 | 2-OH 16:0 | 1±0.5 |
| 19.44 | fatty alcohol 18:0 | 2±0.5 |
| 20.94 | fatty alcohol 19:0 | 2±1 |
| 22.35 | 20:0 | 2±0.5 |
| 25.57 | 22:0 | 2±1 |
| 25.92 | fatty alcohol 22:0 | 0.5±0.2 |
| 28.41 | 2-OH 22:0 | 0.5±0.3 |
| 28.54 | 24:0 | 2.5±0.5 |
| 29.79 | 2-OH 23:0 | 0.5 |
| 31.12 | 2-OH 24:0 | 2.5±0.5 |
| 31.29 | 26:0 | 0.5 |

**Table S2**. The main fatty acid (%) in *Convolvulus arvensis* L.

| **Retention time** | **Fatty acid** | **Relative content (%)** |
| --- | --- | --- |
| 10.44 | 14:0 | 1 |
| 14.89 | 16:1 | 4±0.5 |
| 14.99 | 16:0 | 32±3 |
| 16.96 | 17:0 | 1±0.5 |
| 17.86 | 2-OH 15:0 | 2 |
| 18.85 | 18:0 | 10±1.5 |
| 18.97 | 2-OH 16:0 | 1 |
| 19.21 | 11-OH 16:0 | 10±1.5 |
| 19.38 | unknown | 7±1 |
| 19.44 | fatty alcohol 18:0 | 2 |
| 20.81 | 11-OH 17:0 | 1 |
| 22.35 | 20:0 | 1 |
| 25.57 | 22:0 | 2±0.6 |
| 26.99 | 2-OH 21:0 | 1 |
| 28.41 | 2-OH 22:0 | 3 |
| 28.54 | 24:0 | 2 |
| 29.50 | unknown | 6±1 |
| 29.79 | 2-OH 23:0 | 1 |
| 31.12 | 2-OH 24:0 | 4±0.6 |
| 31.29 | 26:0 | 1 |
| 32.42 | 2-OH 25:0 | 1 |
| 33.67 | 2-OH 26:0 | 1±0.5 |
| 33.89 | 28:0 | 1 |
| 34.02 | fatty alcohol 28:0 | 1 |
| 36.40 | fatty alcohol 30:0 | 4±1 |
